# Galangin alleviates rheumatoid arthritis in rats by downregulating the phosphatidylinositol 3-kinase/protein kinase B signaling pathway

**DOI:** 10.1080/21655979.2022.2062969

**Published:** 2022-04-29

**Authors:** Xiongwei Deng, Hailang Le, Taohong Wan, Meizhi Weng, Yongzhen Tan

**Affiliations:** a Orthopedics and Traumatology, Nanchang Hongdu Hospital of Traditional Chinese Medicine, Nanchang, Jiangxi, China; b College of Traditional Chinese Medicine, Jiangxi University of Traditional Chinese Medicine, Nanchang, Jiangxi, China; c Department of Traditional Chinese Medicine, The Second Affiliated Hospital of Guangzhou Medical University, Guangzhou, Guangdong Province, China

**Keywords:** Rheumatoid arthritis, galangin, PI3K/AKT, cell death

## Abstract

Rheumatoid arthritis (RA) is a chronic autoimmune disease that greatly affect patients’ quality of life. Galangin extract is renowned for its anti-proliferative and anti-oxidative characteristics. However, galangin cytotoxicity studies are presently inadequate. We aimed to investigate the therapeutic potential of galangin on RA by investigating the PI3K/AKT signaling pathway.Fibroblast-like synovial cells (FLSs) were exposed to lipopolysaccharide (LPS) to establish an RA model *in vitro*. An ELISA assay was used to detect the levels of IL-1β, TNF-α, and IL-6. Cell viability and apoptosis were determined by CCK8/EdU and flow cytometry assays. A western blot assay was used to analyze the protein expression levels. An RA rat model was established to evaluate the function of galangin through histopathological examination. Our results found that galangin induced apoptosis, inhibited cell proliferation, and increased cell invasion of rheumatoid arthritis fibroblast-like synovial cells (RAFLSs). Galangin inactivated the PI3K/AKT signaling pathway and the inflammatory response. An agonist of PI3K signaling, 740Y‐P, restored the cellular functions of RAFLSs. Moreover, galangin suppressed the development of RA *in vivo*. Galangin effected its anti-arthritic influence through the PI3K/AKT signaling pathway. Galangin has potential as an alternative treatment for RA.

## Graphical Abstract

**Figure:**
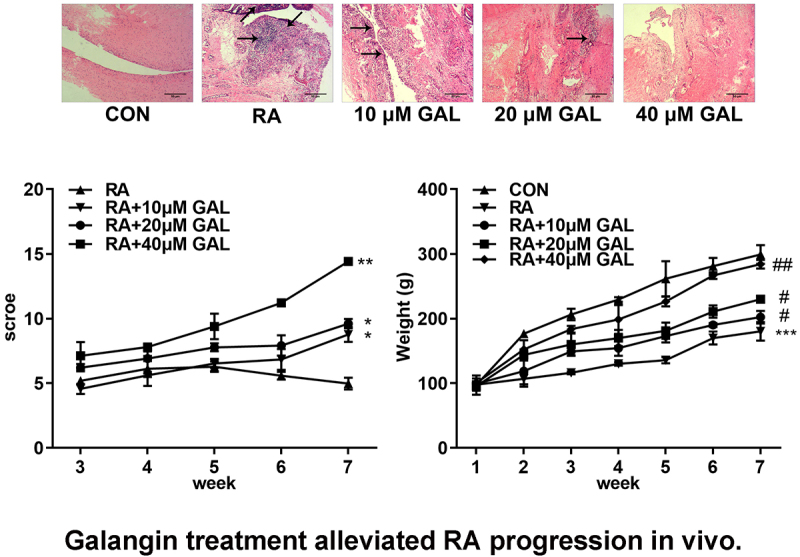

## Highlights

1. Galangin inhibited the proliferation, invasion, and migration of RAFLSs.
2. Galangin treatment alleviated RA progression in vivo.
3. The activation of PI3K/AKT/mTOR signaling promoted the development of RA

## Introduction

Rheumatoid arthritis (RA) is an inflammatory autoimmune disease that affects the joints and eventually leads to joint deformities [1,2]. RA is a high risk health issue globally [3]. To date, RA treatment focuses only on pain relief and anti-inflammation medication to improve patients’ quality of life [4]. The primary parts of RA pathogenesis are the fibroblast-like synoviocytes in the joint synovial membrane [5]. The synovial membrane is vital in reducing the friction between the joint cartilages and to provide nutrients [6]. The physiology of the fibroblast-like synoviocytes is significantly changed during RA progression, which presents as the loss of contact inhibition properties and over-proliferation events. Moreover, the fibroblast-like synoviocytes secrete various pro-inflammatory cytokines, including tumor necrosis factor (TNF)-α and interleukin (IL)-1β, which cause inflammation of the synovium, leading to the joints’ destruction [5–7]. There is, therefore, an urgent need to develop alternative drugs for RA.

RA is an auto-immune disorder. Recently, the PI3K signaling pathway has been proposed as a vital contributor to RA and psoriatic arthritis, in which irregular survival of synovial fibroblasts, activated immune cells, monocytes, macrophages, and dendritic cells are conspicuously associated with the abnormal growth of cancer cells. Recent studies have indicated that targeting the PI3K signaling pathway through the use of small molecules may be a useful immune-mediated arthritis treatment [8].

The natural flavonoid galangin is extracted from the roots of *Alpinia officinarum*. In South Africa, this herb is used to treat infection [9]. Galangin is renowned for its multiple beneficial properties, including anti-proliferative, anti-oxidative, cardioprotective, and immunoprotective traits [10]. The anti-inflammatory properties of galangin have attracted the attention of scientists in the field of RA [11–13].

While the effectiveness of galangin has been previously demonstrated in the treatment of RA [14,15], the present study aimed to investigate the potential mechanism of galangin in LPS-treated rheumatoid arthritis fibroblast-like synovial cells (RAFLSs) *in vivo* in terms of the mTOR/PI3K/AKT signaling pathway. We hypothesized that galangin effected its anti-arthritic influence through the PI3K/AKT signaling pathway.

## Materials and methods

### Cell culture

FLSs were provided by the Cell Bank of Chinese Academy of Sciences (Shanghai, China) and cultured in Dulbecco’s modified Eagle’s medium (Thermo Scientific, Inc., USA) containing 10% fetal calf serum (Sigma-Aldrich, Germany) and supplied with 100 U/mL penicillin and 100 µg/mL streptomycin. We used cells from the logarithmic growth phase.

Cells were treated with lipopolysaccharide (LPS, 100 ng/mL) to establish a RA model in vitro. After that, galangin (10, 20, 40, 80 and 160 µM) and/or PI3K agonist 740Y‐P (30 µg/mL) were used to treat the cells.

### ELISA

According to a previous study [16], RAFLSs were homogenized in PBS and centrifuged at 1,000 × *g* for 15 min at 4°C. The supernatant was subjected to ELISA detection of IL-1β, TNF-α, and IL-6 using ELISA kits (MSK Bio) following the manufacturers’ protocols. We detected the absorbance of the samples at 450 nm using a microplate reader (Thermo Fisher Scientific, Inc.).

### Western blotting

According to a previous study [17], RAFLS total protein was lysed in lysis buffer (Beyotime Institute of Biotechnology, Haimen, China), and the protein concentration was determined using BCA assay. Equal protein amounts were loaded and separated by 10% SDS-PAGE and transferred onto PVDF membranes. The membranes were incubated with primary antibodies against the target proteins at 4°C overnight, followed by incubation with HRP-conjugated goat anti-rabbit IgG and fluorescent-labeled secondary antibodies (GenScript) at 37°C for one hour. After incubation with secondary antibodies, enhanced chemiluminescence solution (Solarbio) was added, and the blots were exposed to x-rays on films in a dark room.

### TUNEL assay

TUNEL staining kit (Yeasen, Shanghai, China) was used for cell death determination. After washing, the cells were fixed with paraformaldehyde for 20 min. Permeabilization was conducted using 0.2% Triton X-100 at 25°C for 5 min. Then the cells were incubated with TdT reaction buffer containing Alexa Fluor 488–12-dUTP Labeling Mix at 37°C for 1 h. Finally, stained cells were visualized and photographed using a fluorescence microscopy (Olympus, Tokyo, Japan).

### Flow cytometry

The flow cytometry analysis was performed as previously described [18]. Briefly, apoptotic cells were stained using an Annexin V-FITC Apoptosis Detection Kit (CA1020; Beijing Solarbio Science & Technology Co., Ltd.) and detected using an Attune NxT Flow Cytometer and its supporting software (Thermo Fisher Inc.). To each well of the 6-well plate, 5 μL of annexin V-FITC was added, and the cells were resuspended at a density of 1 × 10^6^ mL.

### CCK8 and EdU assay

We evaluated the galangin cytotoxicity using the Cell Counting Kit 8 (CCK8, Abcam) as previously described [19]. In brief, the cells were seeded into 96-well plates in the presence of various galangin concentrations for 24, 48, and 72 h. Ten μL of CCK-8 reagent was added to each well before incubation for two hours. The absorbance was measured at 450 nm. The EdU assay was performed following the manufacturer’s protocol (Abcam).

### Colony formation assay

In brief, RAFLSs were seeded into 6-well plates and culture for 7 days with the culture medium refreshing every 2 days. Thereafter, the cells have been stained by crystal violet (0.1%) for ten minutes. Colonies have been observed and counted by a microscope (Nikon, Tokyo, Japan).

### Animal modeling and histopathological examination

We established an RA animal model with twenty 4-week-old Sprague-Dawley rats. The rats were randomly separated into four groups: RA, RA+10 μg galangin group, and RA+20 μg galangin group, RA+40 μg galangin group. All rats were hypodermically injected with 100 mg chicken collagen II, emulsified 1:1 in complete Freund’s adjuvant. The rats were injected with galangin for 4 weeks. From the 21^st^ day after the first injection, the degree of arthritis was evaluated every 3 days by two observers in a blinded manner. Each paw of the rats was scored for clinical arthritis from 0 to 4 – a maximum score of 16 per mouse – as follows: 0, typical; 1, redness and swelling was apparent in the ankle or one finger; 2, two joints were involved; 3, three or more joints were involved; 4, severe arthritis was apparent in the entire paw and all fingers. The rats were sacrificed on the 49^th^ day and the joint synovial tissues were collected.

For hematoxylin and eosin (HE) staining, the tissues were immersed in 4% paraformaldehyde for 48 h and embedded in paraffin. The specific experimental steps of HE) staining refer to the previous study [20]. Briefly, The sections (5 μm) were stained with either Hematoxylin and Eosin using standard procedures. The images were acquired under a 200× magnification using the light microscopy mode of a fluorescence microscope. The rats were weighed every 7 days and monitored for 7 weeks.

### Ethics approval

This study was approved by Ethics Committee of Nanchang Hongdu Hospital of Traditional Chinese Medicine on September 26th, 2021 (approval number KYKS-2021141).

### Statistical analysis

GraphPad Prism (version 8.2.1.441, GraphPad Software Inc.) was used to analyze the data. The results were expressed as means ± SD. Student’s *t*-test was performed on comparisons between 2 groups, whereas the analysis of variance was used for comparisons among multiple groups. *P* < 0.05 was considered statistically significant.

## Results

In this study, galangin plays a beneficial role in RA. Galangin suppressed the inflammation response, proliferation, migration and invasion, and promoted the apoptosis of RAFLSs. Moreover, galangin improved the histology of arthritis. Galangin effected its anti-arthritic influence through the PI3K/AKT signaling pathway. Galangin has potential as an alternative treatment for RA.

### The effects of galangin on RAFLSs

**Figure 1.**
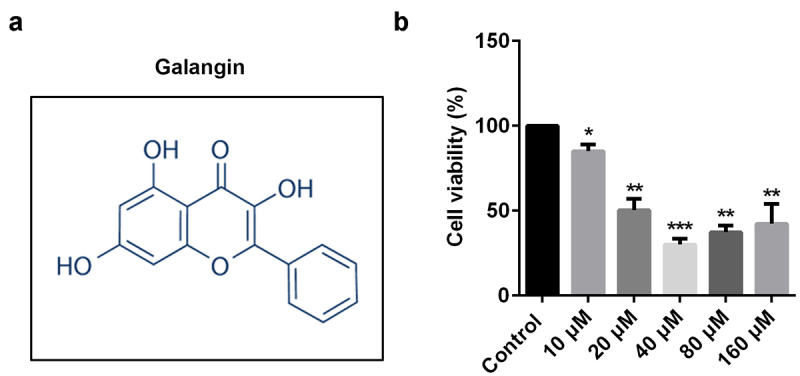
Galangin affects rheumatoid arthritis (RA) cell protein expression. (a) The molecular structure of galangin. (b) The inhibitory effects of galangin on cell viability of the rheumatoid arthritis fibroblast-like synovial cells (RAFLSs). *P < 0.05, **P < 0.01, ***P < 0.001 vs. control.

Various studies reveal that galangin plays a protective role in RA. Figure 1(a) shows the molecular structure of galangin. RAFLSs were exposed to 10, 20, 40, 80, and 160 µM of galangin (Figure 1(b)); however, cell viability was significantly increased in the 80 and 160 µM groups. Accordingly, 10, 20, and 40 µM concentrations of galangin were used in the following experiment. These results indicated that 10, 20, and 40 µM concentrations of galangin effectively inhibited the RAFLSs growth.

### Galangin inhibited the proliferation, invasion, and migration of RAFLSs

**Figure 2.**
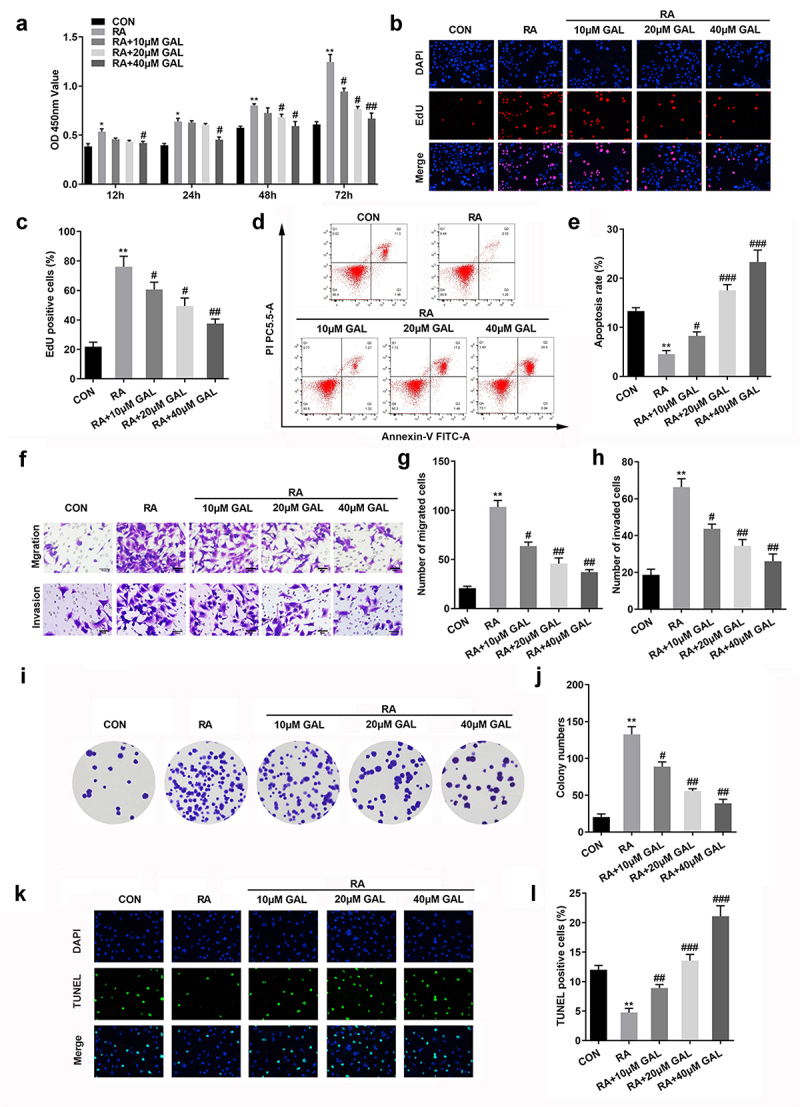
Galangin enhances cell apoptosis rate, inhibits RA cell proliferation, invasion, and migration. (a) CCK8 and (b-c) EdU assays showed that galangin markedly restrained RAFLSs cell viability and proliferation. (d-e) Cell apoptosis was analyzed by flow-cytometry assay. Transwell migration and invasion assays (f-h) were used to detect cell invasion and migration. (i-j) Clone formation of RAFLSs. (k-l) TUNEL staining of the RAFLSs. *P < 0.05, **P < 0.01, vs. CON; ^#^P < 0.05, ^##^P < 0.01, ^###^P < 0.001, vs. RA. CON, control; RA, rheumatoid arthritis fibroblast-like synovial cells; GAL, galangin.

To further investigate the effects of galangin on RA, FLSs were cultured with 10, 20, and 40 µM of galangin. As shown in Figure 2(a), LPS treatment increased the cell viability of RAFLSs, which was alleviated by galangin in a time- and dose-dependent manner. Moreover, LPS treatment significantly increased the cell proliferation and decreased the cell apoptosis, while galangin treatment antagonized the effects of LPS on the proliferation of RAFLSs and promoted the apoptosis of RAFLSs (Figure 2(b–e)). Additionally, LPS treatment also significantly increased the the migration and invasion ability of RAFLSs, while galangin treatment significantly suppressed the migration and invasion ability of RAFLSs induced by LPS (Figure 2(f–h)). Besides, LPS treatment significantly increased the colony formation of the RAFLSs, while galangin treatment decreased it (Figure 2(i,j)). TUNEL staining indicated that LPS treatment significantly decreased the cell death of the RAFLSs, while galangin treatment increased it (Figure 2(k,l)). These results indicated that galangin played a inhibitory role in the proliferation, migration and invasion of the RAFLSs, and played a promoting role in the apoptosis of the RAFLSs.

### Galangin treatment alleviated RA progression in vivo

**Figure 3.**
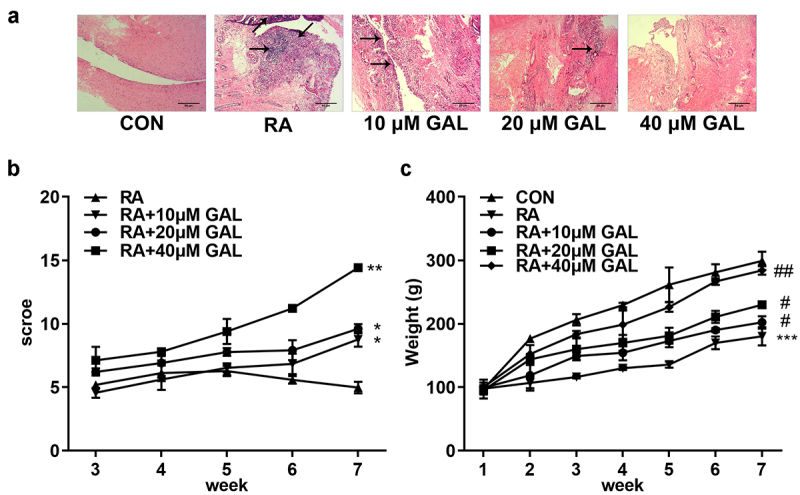
Galangin effectively ameliorated RA of the rat models. (a) Histological examination images of the fibroblast-like synoviocytes from the RA rats. (b) RA scores of the rats treated with different galangin concentrations. (c) Weight changes of the galangin-treated RA rats. *P < 0.05, **P < 0.01, ***P < 0.001, vs. CON; ^#^P < 0.05, ^##^P < 0.01, vs. RA. CON, control; RA, rheumatic arthritis; GAL, galangin.

*In vivo* assays were performed to further verify the effects of galangin on RA. As shown in Figure 3(a), galangin improved bone/cartilage destruction, synovial hyperplasia, and pannus formation in the RA rats. Moreover, galangin improved histological scores (Figure 3(b)) and increased the body weight of rats (Figure 3(c)). With the increase of the concentration of galangin, its therapeutic effect is getting better and better. These results indicated that galangin effectively relieved tha RA progression *in vivo*.

### Galangin inhibited the RA-related cytokines TNF-α, IL-1β, and IL-6, and downregulated the PI3K/AKT/mTOR signaling pathway in vivo

**Figure 4.**
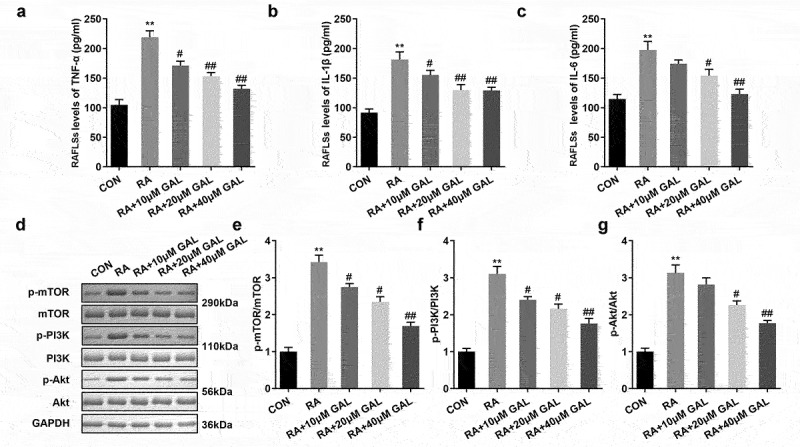
Galangin suppresses inflammatory cytokine secretion and the PI3K/AKT/mTOR pathway in RA rats. The concentrations of (a) TNF-α, (b) IL-1β, and (c) IL-6 in the peripheral blood of the RA rats. Galangin decreased the (d) p-mTOR, (e) p-PI3K and (f) p-AKT in the joint synovial tissues of the RA rats. **P < 0.01, vs. CON; ^#^P < 0.05, ^##^P < 0.01, vs. RA; CON, control; RA, rheumatic arthritis; GAL, galangin.

We further investigated the cytotoxic effect of galangin *in vivo*. In the RA rats, the TNF-α, IL-1β, and IL-6 levels were significantly increased in the peripheral blood, while Galangin substantially inhibited levels of the peripheral blood cytokines TNF-α, IL-1β, and IL-6 in a dose-dependent manner (Figure 4(a–c)). Also, pPI3K, pAKT, and pmTOR protein levels were significantly increased in the RA rats. While galangin treatment downregulated the PI3K/AKT signaling pathway, as indicated by the inhibition of pPI3K, pAKT, and pmTOR protein levels (Figure 4(d–f)). These results indicated that galangin relieved the RA progression through suppressing the inflammatory reaction and inactivating the PI3K/AKT/mTOR signaling pathway

### The activation of PI3K/AKT/mTOR signaling promoted the development of RA

**Figure 5.**
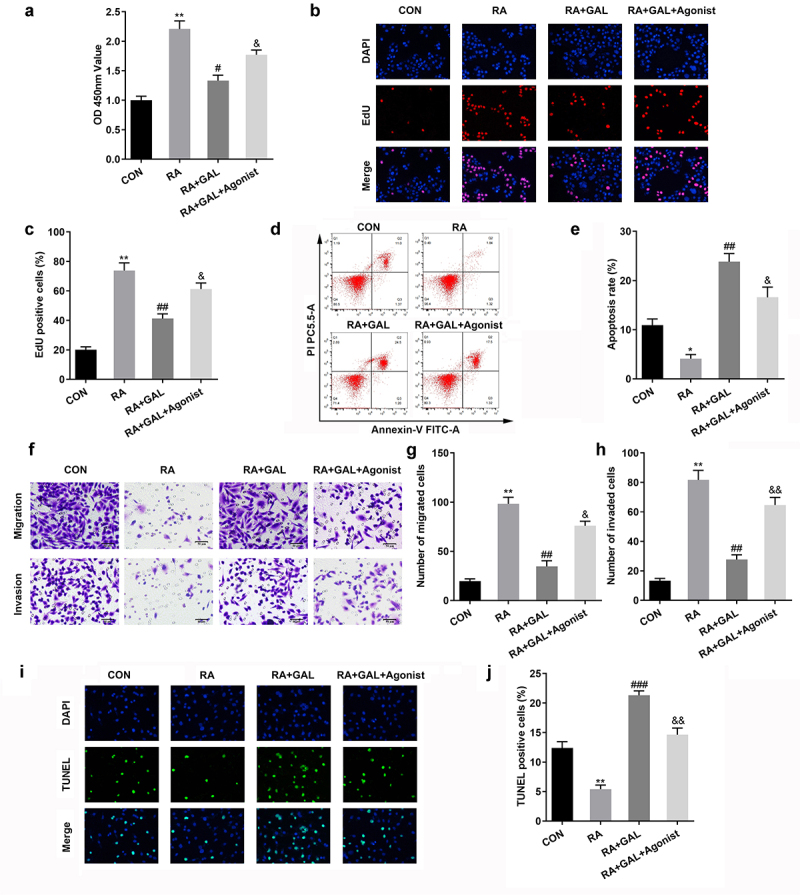
The activation of PI3K/AKT/mTOR signaling restored the cellular function of RAFLSs. (a) CCK8 and (b-c) EdU assays showed 740Y-P markedly increased the cell viability and proliferation of the RAFLSs. (d-e) Cell apoptosis of the RAFLSs was analyzed by flow cytometry assay after. (f-h) Transwell assay was used to detect cell invasion and migration. (i-j) TUNEL staining was used to detect the cell death. *P < 0.05, **P < 0.01, vs. CON; ^#^P < 0.05, ^##^P < 0.01, vs. RA. ^&^P < 0.05, ^&&^P < 0.05, vs. RA+GAL; CON, control; RA, rheumatic arthritis; GAL, galangin.

PI3K/AKT/mTOR signaling stimulates the proliferation and inflammatory response of RAFLSs. 740Y-P, an agonist of PI3K/AKT/mTOR signaling, significantly increased the cell viability and proliferation of the galangin treated RAFLSs (Figure 5(a-c)). Additionally, the cell apoptosis of the galangin treated RAFLSs was significantly decreased after 740Y-P treatment (Figure 5(d-e)). Besides, the cell migration and invasion of the galangin treated RAFLSs was significantly increased after 740Y-P treatment (Figure 5(f-h)). TUNEL staining showed that the cell death of the galangin treated RAFLSs was significantly decreased after 740Y-P treatment (Figure 5(i,j)). These results implied that activating the PI3K/AKT/mTOR signaling pathway neutralized the galangin effects on the cell growth of the RAFLSs.

### 740Y-P promoted inflammatory response and activated PI3K signaling

**Figure 6.**
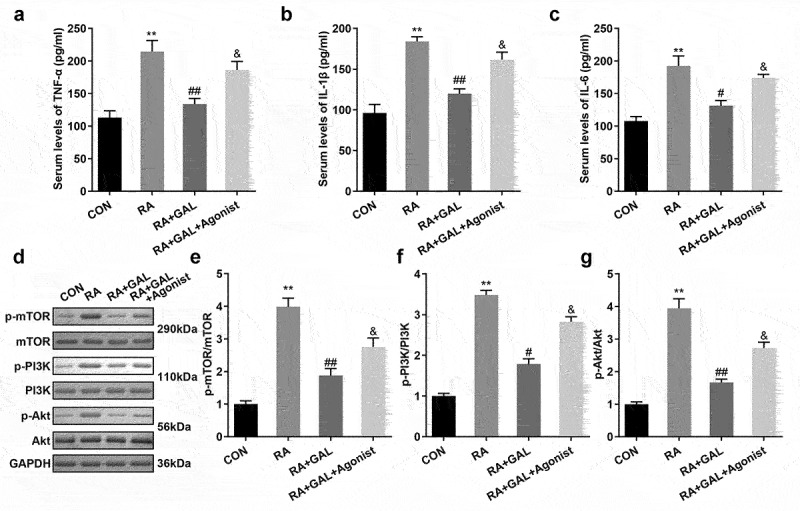
740Y-P promoted inflammatory response and activated PI3K signaling. The concentrations of (a) TNF-α, (b) IL-1β, and (c) IL-6 in the peripheral blood of the RA rats. 740Y-P increased the (d) p-mTOR, (e) p-PI3K and (f) p-AKT in the joint synovial tissues of the RA rats after galangin treatment. **P < 0.01, vs. CON; ^#^P < 0.05, ^##^P < 0.01, vs. RA; ^&^P < 0.05, vs. RA+GAL group. CON, control; RA, rheumatic arthritis; GAL, galangin.

To further investigate PI3K signaling on RA, The RA rats were treated with 740Y-P. As shown in Figure 6, 740Y-P significantly increased release of the peripheral blood cytokines TNF-α, IL-1β, and IL-6 in the galangin treated RA rats (Figure 6(a–c)). Moreover, 740Y-P activated the PI3K/AKT signaling pathway, as indicated by the up-regulation of pPI3K, pAKT, and pmTOR protein levels (Figure 6(d–f)). These results implied that activating the PI3K/AKT/mTOR signaling pathway neutralized the galangin effects on the inflammatory reaction of the RAFLSs.

## Discussion

RA is a result of systemic inflammatory and immune turmoil [21,22]. In this study, galangin plays a beneficial role in RA. Galangin suppressed the inflammation response, proliferation, migration and invasion, and promoted the apoptosis of RAFLSs. Moreover, galangin improved the histology of arthritis.

Galangin, with its anti-proliferative and immunoprotective properties, is widely used in the treatment of bone disorders. For instance, galangin promotes dendritic cell proliferation, which induces the differentiation of immunosuppressive regulatory T cells [23]. Moreover, galangin impedes osteoclastogenesis by inactivating NF-κB, which transcriptionally activates inflammatory signaling [24]. This anti-inflammatory effect may be a promising strategy for RA treatment. In this study, galangin suppressed the proliferation and promoted the apoptosis of RAFLSs. Moreover, galangin improved the histology of arthritis *in vivo*. The progression of RA was accompanied by the proliferation of RAFLSs, which secreted pro-inflammatory cytokines [5–7,25–28]. Previous studies have established that the development of the osteoclast-rich portion of the synovial membrane (pannus) of RA synovial joints increased the tumor risk [29]. Hence, galangin may improve the microenvironment of RA and may be a promising strategy for treating or managing RA.

The PI3K/AKT signaling pathway regulatory function in cancer cells seems to correlate with RA, characterized by the aberrant survival of activated cells arising from dysfunctional innate and adaptive immune systems and by the proliferation of activated synovial-tissue fibroblasts [8]. Accordingly, the abnormal survival of cells in the RA joints was attributed to the altered PI3K signaling pathway. This suggests that the PI3K signaling members are worthy targets for developing small molecular inhibitors [30] as well as developing PI3K inhibitors to treat other autoimmune diseases, such as systemic lupus erythematosus [31], Crohn’s disease [32] and antiphospholipid syndrome [33]. In this study, galangin inactivated PI3K signaling; activation of PI3K signaling alleviated the effects of galangin and promoted/restored the cellular functions of RAFLSs and promoted inflammatory response. These results suggest that galangin may suppress the development of RA by inactivating PI3K signaling.

## Conclusion

In conclusion, galangin suppressed the development of RA via the PI3K/AKT signaling pathway. These results reveal a novel mechanism of galangin and indicate that it may be a feasible RA treatment.
